# Calibration of Radon-222 Reference Instrument in Sweden

**DOI:** 10.6028/jres.095.011

**Published:** 1990

**Authors:** R. Falk, H. Möre, L. Nyblom

**Affiliations:** National Institute of Radiation Protection, Stockholm, Sweden

**Keywords:** alpha-spectrometry, calibration, intercomparison, radon measurement, reference instrument, surface barrier detector

## Abstract

A simple procedure to calibrate and characterize a recently developed radon-222 reference instrument is described. The system, which is now used as the official national Swedish reference, is quick and easy to use. Systematic as well as random errors are smaller than in an earlier system and compare well with other systems, as has been shown in a number of international intercomparison measurements.

## 1. Introduction

In the early 1970s it was recognized that radon in indoor air in Sweden was the source of a significant radiation dose to the population. As the dose assessment is based on measurements of radon and its decay products the need for coordinated calibration of measuring instruments became obvious.

A technique for calibration of a reference instrument for radon measurement was developed and adopted. The reference instrument used at that time was an ionization chamber with a measuring volume of 18 L [1]. In 1987 the ionization chamber was replaced by an instrument with higher sensitivity and accuracy. This instrument is based on *α*-spectrometric measurements of radon daughters electrostatically collected on a surface barrier detector. The instrument is designed according to the principles described by Wicke [2], and has a measuring volume of 10.8 L.

The calibration of both instruments is traceable directly to the National Institute of Standards and Technology (NIST) through the use of its Stan-dard Reference Material Radium-226. The reference source consists of the NIST radium solution in a set of glass containers.

To collect and transport radon samples for calibration or measurement, a steel container of volume 4.74 L is used. The same transfer and measuring procedure is used both for calibration and for the determination of radon concentrations in air samples. A description of the procedures used, together with estimates of the uncertainties associated with the calibration technique, measuring method and instrument used are given below.

## 2. Materials and Methods

### 2.1 The Reference Source

**Figure 1:**
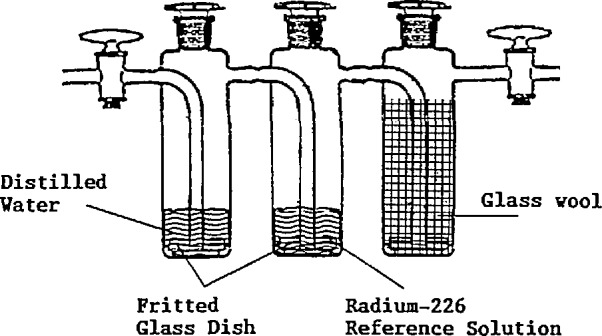
The reference source.

Figure 1 is a schematic diagram of the radon reference bubbler. The three gas washing bottles are made of glass and fused to form a single unit. Also the stoppers and the valves are made of glass to ensure a radon-tight system. The first gas washing bottle contains distilled water, the second the NIST Radium-226 Standard Reference Material and the third glass wool. The total volume of the system is approximately 500 cm^3^ and the volume of the reference solution is 54 cm^3^.

When flushing the system with air, the air is first humified in the water bottle, then passes through the reference radium solution and finally through the glass wool, where water droplets will be removed from the radon-laden air stream.

Radon-free air, from a cylinder of aged air kept for the purpose, is used to flush the reference bubbler to remove accumulated radon. A volume of aged air of 4.74 L is used and complete removal of radon is achieved. A second flushing, immediately after the first, with the same volume of aged air removes less than 0.06% of the radon removed initially.

The build-up of radon-222 in the system is determined by the radium-226 activity of the reference solution and the decay constant of radon-222. For calibration purposes build-up times between 7 h and 14 d have been used. Shorter build-up times can be used but this increases the uncertainty in the radon-222 activity accumulated. At build-up times longer than two weeks, any small leakage of radon from the glass bottles will also influence the uncertainty of the calibration. Leakage of radon from the reference source has been checked and the uncertainty in the accumulated radon-222 due to leakage is less than 0.2%.

The NIST Radium-226 Standard Reference Material is certified to contain 31.33 Bq as of April 1978, with an uncertainty of 1.53%. The uncertainty in the radon activity removed from the bubbler is calculated to be 1.6%, which is the sum of the uncertainty in the reference activity and the removal error.

### 2.2 The Transfer Container

A commercially available steel container normally used for propane gas is used for sampling and calibration. Its volume has been determined by two independent methods.

The first method is based on comparison with the known volume of another metal container. The design of this second container is chosen so that its volume can be calculated accurately from its dimensions. The comparison of the two volumes is by an air-pressure measurement. One of the containers is evacuated to a very low pressure and then connected to the other container which is held at atmospheric pressure. The gas pressure in the containers are monitored before and after the connection. The volume of the “sample and transfer” container from repeated measurements was found to be 4734±3 cm^3^ (1 S.D.). The systematic uncertainty is estimated to be 9 cm^3^ (0.2%).

The second method used for determination of the volume is based on a water displacement technique. The container’s outer volume is determined by measuring the volume of water displaced by the container when it is submerged. The volume of the metal in the container is assessed from the weight of the container and the density of the metal. In this way the volume is found to be 4753 ±19 cm^3^ (±0.4%), total overall error.

From these two determinations the volume of the sample and transfer container is estimated to be 4740 cm^3^ at 23 °C, with a maximum error of 0.5%.

### 2.3 The Reference Instrument

The reference instrument for the determination of radon-222 in air is based on *α*-spectrometric measurements of polonium-218 and polonium-214 electrostatically collected on a surface barrier detector. The instrument is built according to the principles and experiences described by Wicke [2].

**Figure 2:**
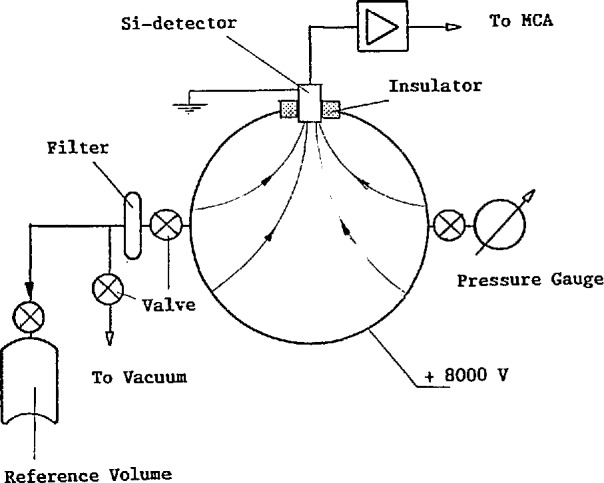
The reference instrument.

Figure 2 is a schematic diagram of the instrument. The sensitive volume of the instrument is a sphere of aluminum with a volume of 10 810 cm^3^ that contains the radon sample to be analyzed. In the surface of the sphere is a surface barrier detector of 150 mm^2^ active area which is electrically isolated from the metal sphere.

A potential of 8 kV is applied between the detector surface and the metal sphere generating an electrostatic field that moves the charged polonium-218 to the detector surface. The decay of radon-222 within the sphere will generate polonium-218 at a rate proportional to the radon-222 activity. A build-up of polonium-218 activity on the detector surface takes place and after approximately 20 min collection a steady state is reached. The polonium-218 activity detected by the α-detector is then proportional to the radon-222 activity in the sphere.

To obtain accurate results from the instrument, the collection efficiency of polonium-218 on the detector surface and the α-counting efficiency must be stable.

The volume of the detector sphere is of no importance for the calibration and measurements but must be constant. Great efforts have been made to ensure the air tightness of the sphere.

To estimate the fraction of the polonium-218 atoms formed in the sphere that are collected on the detector surface, however, the volume must be known. From the geometrical dimensions, the volume has been determined as 10 790 cm^3^ and from pressure comparison measurements with a known volume as 10 820 cm^3^. A value of 10 810 cm^3^ is adopted with a maximum error of ±100 cm^3^ (±1%).

The collection of polonium-218 on the detector surface and the counting efficiency of the *α*-particles from polonium-218 depend on a number of parameters such as the geometry and strength of the electric field, and the air pressure and humidity within the detector volume.

Parameters have been chosen so as to keep the parameter sensitivity as low as possible. The following apply to the most important parameters.

- The geometrical position of the detector affects the form and strength of the electric field between the chamber walls and the detector surface.
- The voltage generating the electric field and the air pressure inside the counting volume are also of concern. We have chosen 600 mbar working pressure and a high voltage of 8 kV. This gives a good margin against electrical breakdown while the collection efficiency as a function of the voltage applied is well within a flat plateau.
- The collection efficiency is affected by the humidity within the counting volume. To avoid this problem the air sample is dried during collection into the sample or transfer container. Different techniques have been tested but sampling through a drying agent (magnesium perclorate) fulfills the requirements. The sample has a humidity of less than 1 *μ*g/L, corresponding to a dew point of less than −30°C.

**Figure 3:**
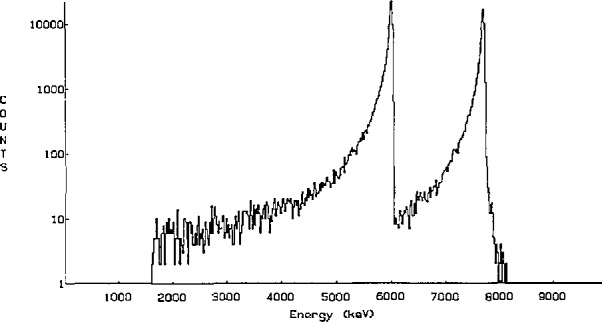
The d-spectrum from the reference instrument.

The surface barrier detector is connected to a multi-channel analyzer. Figure 3 shows a typical *α*-spectrum. The energy resolution of the detector separates almost completely the *α*-particles from polonium-218 and polonium-214. The *α*-particle count-rate within the energy band 4.34 to 6.16 MeV is used for evaluation. The affect of electronic instability has been studied and is negligible. For measurement of low radon-222 concentrations, correction for background counts is needed. The background with radon-free air corresponds to a radon-222 concentration of 0.6 Bq/m^3^.

Adsorption of radon onto the inner surface of the sphere has been observed. As the sphere is made of aluminum the metal surface will be covered with a thin layer of aluminum oxide. It is reasonable to assume that the oxide layer causes this effect, as it is known that aluminum oxide adsorbs other gases. The effect is small and reproducible. A reduction in the radon content in the sphere of 0.1 % per h is found and corrected for.

### 2.4 Calibration Procedure

#### 2.4.1 Preparation of the Radon-222 Reference Concentration

- The reference source is flushed with 4.74 L of radon-free air and the valves of the reference source are then closed. The date and time are noted and the source is left undisturbed for the build-up of radon.
- The transfer container and the detector volume of the reference instrument are evacuated to less than 1 mbar.
- The transfer container is connected to the outlet of the reference source via a short tube containing the drying agent. A plastic bag filled with radon free air is connected to the inlet of the reference source. When the valves of the container and the reference source are opened, the radon is sucked into the container. The procedure is standardized to take 5 min.
- The valves are closed and time, temperature and ambient air pressure are recorded.
- As the volume of the transfer container and the radon-222 activity are known, the air concentration of radon-222 in Bq/m^3^ of dry air in the container can be calculated.

#### 2.4.2 The Transfer of Radon to the Reference Instrument

- The transfer container is connected to the reference instrument by a tube. A particle filter is inline at the entrance to the detector. An additional tube, provided with a valve, is connected by a T-piece to the tube between the container and the instrument.
- The above mentioned tubes are evacuated and the valve on the tube is closed leaving the connection between container and instrument under vacuum.
- The valve at the instrument inlet is opened and when the valve on the transfer container is opened the pressure difference will transfer about 70% of the radon in the container to the detector volume.
- The two volumes should be at the same temperature during the transfer procedure.
- The procedure takes about 10 min. The last 5 min establishes pressure equilibrium between the two connected volumes. The air pressure inside the detector will be approximately 300 mbar.
- There is no need to know exactly the amount of radon transferred. The relative amount, however, must be the same on every occasion. This depends only on the volumes of the transfer container and the detector.
- Time and temperature are recorded when the transfer is completed.
- Radon-free air is then added to the detector until the air pressure reaches 600 mbar. The valve at the detector is then closed.
- The electrostatic field for the detector is switched on and 30 min allowed to elapse to establish a steady state activity of polonium-218 on the detector surface.

#### 2.4.3 Counting and Evaluation

- *α*-spectrometric counting is carried out for a sufficient time to ensure that adequate counting statistics are achieved.
- The mean net count rate of *α*-particles from polonium-218 within the energy band 4.34 to 6.16 MeV is calculated. Corrections for radioactive decay and adsorption of radon are applied and the calibration factor in Bq/m^3^ of radon in dry air per count/s is evaluated.

### 2.5 Procedure for Determination of Radon Concentration in Air Samples

#### 2.5.1 Sample Collection

- The same, or identical, transfer containers are used for sample collection and calibration purposes.
- The container is evacuated to less than 1 mbar and a short tube containing the drying agent is connected to the inlet of the container.
- The sample is taken by opening the valve on the container. The filling takes 5 min. The valve is left fully open for another 5 min to ensure that pressure equilibrium is reached. The valve is then closed and the temperature, pressure, and humidity of the ambient air as well as the date and time are recorded.
- The sample container now contains dry air at the ambient air pressure. Thus the radon concentration in the container is slightly higher than in the ambient air.

#### 2.5.2 Transfer of Radon

The transfer of radon to the reference instrument is done by the same procedure as is used for calibrations; see section 2.4.2.

#### 2.5.3 Counting and Evaluation

Counting and the evaluation of the spectrum is carried out with the same procedure as is used for calibrations; see section 2.4.3. The radon concentration in dry air is then calculated using the calibration factor, and a correction is applied to compensate for the low humidity in the container. The corrected value will then be the radon concentration in Bq/m^3^ in air, but only for the temperature, pressure, and humidity existing during sampling.

## 3. Calibration Results and Error Calculations

**Figure 4:**
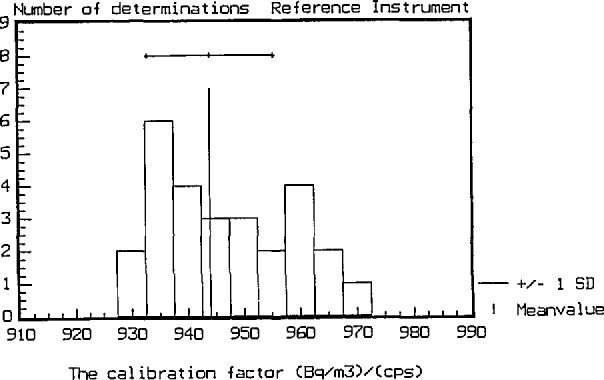
The distribution of the calibration factor.

**Figure 5:**
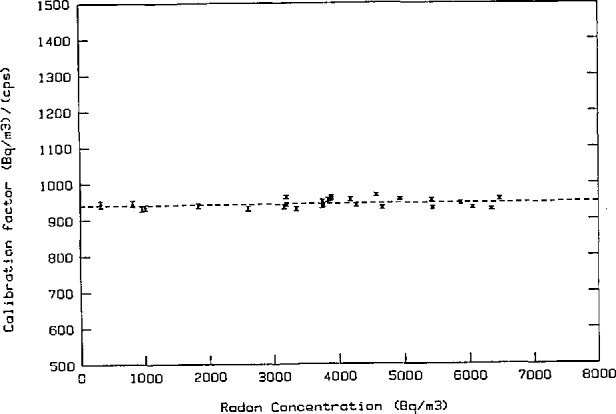
Calibration of radon reference instrument.

Calibration factors from 30 independent calibrations with radon concentrations between 300 to 6500 Bq/m^3^ are summarized in figures 4 and 5. The mean value of the calibration factor is 944 Bq/m^3^ per count/s with a standard deviation (S.D.) of 1.2% and a standard error (S.E.) of 0.2%, which indicates the precision of the calibration factor. The uncertainty arising from counting statistics is 0.6% (1*σ*).

Known systematic errors in the calibration factor stem from the uncertainty in radon activity removed from the bubbler, 1.6%, and the volume of the transfer container 0.5%. Systematic errors due to thermal and pressure disequilibrium during the calibration procedure and uncertainties related to temperature and pressure measurements are estimated to be less than 1%. Thus the total systematic error in the calibration factor is less than 3.1%.

The uncertainty in the calibration factor, calculated as the linear sum of the standard error at 99% confidence level and the maximum likely systematic error, is 0.6% + 3.1% = 3.7%.

The precision of the measurement of radon samples is calculated from the sum of the uncertainty arising from counting statistics and the precision of repeated measurement of calibration samples. The sum of the variances is used to calculate the precision. The precision from repeated measurements of calibration samples is 1.0% excluding counting statistics. Assuming an uncertainty arising from counting statistics of 1.0% (1*σ*), the precision of a measurement will be 1.4% (1*σ*).

For radon sample measurements, systematic errors other than those in the calibration factor are estimated to be at most 0.5%—thus there is a maximum systematic error of 4.2% in these measurements.

## 4. Results from Intercomparison Measurements

The results from the third Commission of the European Communities (CEC) intercomparison of active and passive detectors for the measurement of radon and radon decay products at the National Radiation Protection Board (NRPB), England (1987) [3] and from intercomparison measurements with the Environmental Measurement Laboratory (EML) in New York (1984) [4] are summarized in table 1.

**Figure 6:**
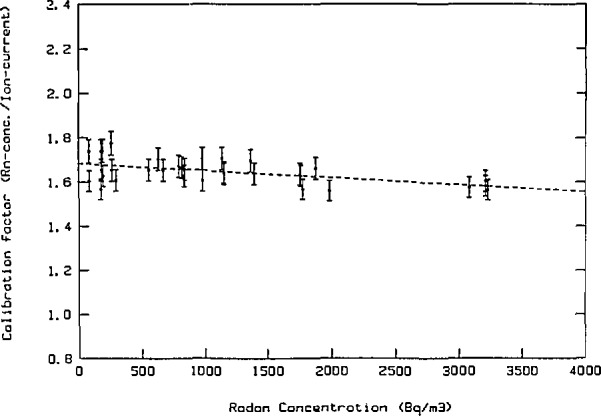
Calibration of radon reference instrument (old).

**Figure 7:**
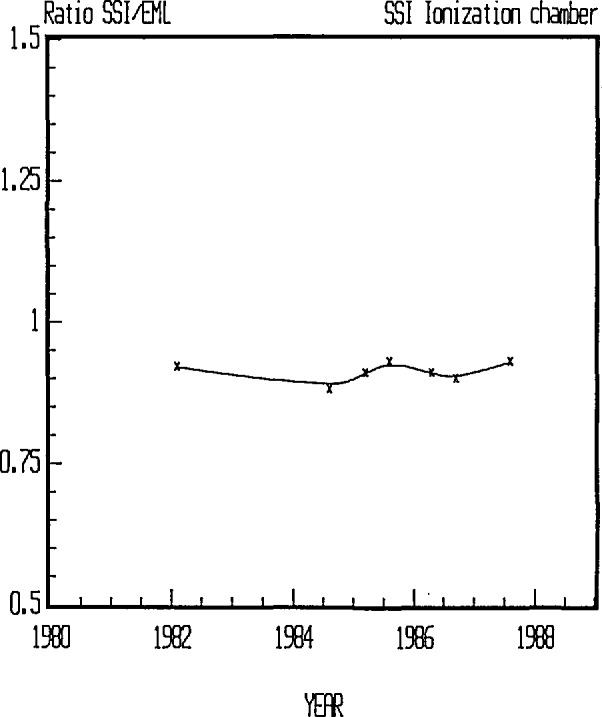
Intercomparison measurements of radon with EML, New York.

Before 1987 an ionization chamber was used as the reference instrument [1]. The calibration procedure used was in principle the same as described above, but the uncertainty obtained with this instrument was larger. Figure 6 shows the results from calibration measurements during the years that the previous instrument was used. Intercomparisons with EML have been performed on seven occasions in the period 1982 to 1987 [4]. The results are summarized in figure 7. Compared to EML our ionization chamber gave as a mean 9*%* lower values. The origin of this difference may be due to larger uncertainties of the instrument used previously and the different radium-226 reference solutions used for calibration at EML and SSI.

## Figures and Tables

**Table 1:** Intercomparison results

Ratio SSI^a^/NRPB	0.95
Ratio SSI/EML	0.96

a SSI=National Institute for Radiation Protection, Stockholm, Sweden.
